# Evaluation of the Safety of Neauvia Stimulate Injectable Product in Patients with Autoimmune Thyroid Diseases Based on Histopathological Examinations and Retrospective Analysis of Medical Records

**DOI:** 10.3390/gels9060440

**Published:** 2023-05-26

**Authors:** Paweł Kubik, Daniela Gallo, Maria Laura Tanda, Jerzy Jankau, Raffaele Rauso, Wojciech Gruszczyński, Aleksandra Pawłowska, Paweł Chrapczyński, Maciej Malinowski, Dariusz Grzanka, Marta Smolińska, Paulina Antosik, Maria-Luiza Piesiaków, Bartłomiej Łukasik, Agnieszka Pawłowska-Kubik, Giorgio Stabile, Stefania Guida, Łukasz Kodłubański, Tom Decates, Nicola Zerbinati

**Affiliations:** 1 K-LAB Badania i Rozwój, 81-312 Gdynia, Poland; 2 Endocrine Unit, Department of Medicine and Surgery, University of Insubria, ASST dei Sette Laghi, 21100 Varese, Italy; 3 Department of Plastic Surgery, Medical University of Gdansk, 80-210 Gdańsk, Poland; 4 Maxillofacial Surgery Unit, University of Campania “Luigi Vanvitelli”, 80138 Naples, Italy; 5 Department of Clinical Pathomorphology, Nicolaus Copernicus University, 85-094 Bydgoszcz, Poland; 6 Medical Department, Matex Lab Switzerland SA, 1228 Geneve, Switzerland; 7 Division of History & Philosophy of Medical Sciences, Medical University of Gdansk, 80-210 Gdańsk, Poland; 8 Department of Clinical Dermatology, Università Vita-Salute San Raffaele, 20132 Milan, Italy; 9 Dermatology Clinic, IRCCS San Raffaele Scientific Institute, 20132 Milan, Italy; 10 Department of Human Rights and Intellectual Property Law, University of Gdansk, 80-309 Gdansk, Poland; 11 Department of Dermatology, Erasmus Medical Center, 3000 Rotterdam, The Netherlands; 12 Dermatologic Unit, Department of Medicine and Surgery, University of Insubria, 21100 Varese, Italy

**Keywords:** Neauvia Stimulate injectable product, hyaluronic acid fillers, autoimmunity, thyroid, polyethylene glycol, NGel, Hashimoto’s

## Abstract

The aim of this study was to test the effect of hyaluronic acid cross-linked with polyethylene glycol containing micronized portions of calcium hydroxyapatite (Neauvia Stimulate) on both local tissue and systemic consequences, which are crucial from the perspective of long-term safety, in patients suffering from Hashimoto’s disease. This most common autoimmune disease is a frequently mentioned contraindication to the use of fillers based on hyaluronic acid as well as biostimulants based on calcium hydroxyapatite. Broad-spectrum aspects of histopathology were analyzed to identify key features of inflammatory infiltration before the procedure and 5, 21, and 150 days after the procedure. A statistically significant effect on the reduction of the intensity of the inflammatory infiltration in the tissue in relation to the state before the procedure was demonstrated, combined with a reduction in the occurrence of both antigen-recognizing (CD4) and cytotoxic (CD8) T lymphocytes. With complete statistical certainty, it was demonstrated that the treatment with Neauvia Stimulate had no effect on the levels of these antibodies. All this corresponds with the risk analysis that showed no alarming symptoms during the time of observation. The choice of hyaluronic acid fillers cross-linked with polyethylene glycol should be considered justified and safe in the case of patients suffering from Hashimoto’s disease.

## 1. Introduction

Soft tissue fillers based on hyaluronic acid are currently one of the leading materials used to rebuild the atrophy of human soft tissues used in aesthetic medicine. Physically, they have the form of a gel with a wide range of densities and viscosities, depending on the intended use (depending on the anatomical area undergoing the procedure, the technique adopted by the doctor performing the procedure, and the individual needs of the patient). The basis for the production of this gel is the cross-linking of hyaluronic acid polymers using several adopted factors. Cross-linking in fillers based on hyaluronic acid is a process that allows the linear chain of hyaluronic acid to be given a three-dimensional structure by creating covalent bonds between the hyaluronic acid and the cross-linking agent, improving the physicochemical properties of hyaluronic acid while maintaining biocompatibility and biological activity [1].

The most commonly used cross-linking agents are butanediol 1,4 diglycidyl ether (BDDE), 1,8-diepoxyoctane (DEO), divinyl sulfone (DVS), and polyethylene glycol diglycidyl ether (PEGDE). Hyaluronic acid cross-linking technologies vary depending on the manufacturer, as well as the degree of cross-linking, the amount of cross-linking agent used, and the concentration of the hyaluronic acid itself. These modifications significantly affect the rheological properties of the gels, which contribute to the aesthetic effect [2].

Soft tissue fillers are becoming increasingly popular in recent years, and because of their increased use, more severe side effects have also become more frequent. Filler-related adverse events are divided into four categories: allergic, infective, late-onset nodules/inflammation, and intravascular events [3,4]. Preoperative evaluations focus on gathering demographic data (race, gender, and generational demands), as well as checking for pre-existing systemic diseases, drugs, and past cosmetic treatments and assessing the state of the patient’s skin. Treatments with hyaluronic acid fillers are contraindicated in patients with autoimmune diseases, such as systemic lupus erythematosus, rheumatoid arthritis, mixed connective tissue disease, and Hashimoto’s thyroiditis [5]. Given the high prevalence of autoimmune disorders, this restriction applies to quite a large group of people. Autoimmune thyroid disorders, which include Hashimoto’s thyroiditis and Graves’ disease, are organ-specific autoimmune disorders whose prevalence reaches 10–12% of the general population [6]. Most autoimmune thyroid disorders are represented by Hashimoto’s disease.

Hashimoto’s thyroiditis was first described in 1912 by Hakaru Hashimoto (1881–1934). In an article published in “Archiv für Klinische Chirurgie” journal, the scientist analyzed histological changes in thyroid tissue based on tissue samples taken from four women. Hashimoto described infiltration of lymphoid and plasma cells, formation of lymphoid follicles with germinal centers, fibrosis, degenerated thyroid epithelial cells, and leukocytes in the lumen. Although Hashimoto pointed out similarities to Mikulitsch’s disease, he clearly established that the described changes indicated a new disease entity, which he called “struma lymphomatosa”. Unfortunately, Hashimoto’s discovery did not receive sufficient attention at the time, and some researchers considered that the “struma lymphomatosa” was an early phase of Riedel’s thyroiditis. Hashimoto’s discovery was forgotten until 1931 when A. Graham and E. P. McCullagh confirmed Hashimoto’s conclusions. Further research in the 1950s led to the concept of organ-specific autoimmune disease, and HT was classified as one of those diseases. More than a hundred years after Hashimoto’s discovery, HT is diagnosed in a growing number of patients but the exact pathogenic mechanisms of HT are still unknown, and the disease itself requires further multidirectional research [7,8,9].

In Hashimoto’s thyroiditis, autoimmune-mediated infiltration of the thyroid gland by innate immune cells (dendritic cells, macrophages, natural killer cells) and lymphocytes and the formation of antithyroid (thyroid peroxidase, TPO, and thyroglobulin, Tg) antibodies and TPO–complement complexes cause progressive fibrosis and damage which eventually results in hypothyroidism [10]. Earlier in the course of the disease, patients are euthyroid, and the diagnosis is based on the detection of circulating anti-TPO and/or anti-Tg antibodies. When the reserve of preformed thyroid hormones is depleted, signs and symptoms of hypothyroidism may occur if treatment is delayed [11].

Hyaluronic acid is one of the major elements in the extracellular matrix (ECM) of vertebrate tissues. It is available in almost all body fluids and tissues, and 50% of the total amount of hyaluronic acid in the human body is located in the skin. The characteristics of this critical unbranched polymer of the ECM are the consistency, biocompatibility, hydrophilicity, and, naturally, the biodegradability. To reduce the fast degradation of the hyaluronic acid solutions, the soft tissue fillers were developed using crosslinked hyaluronic acid. The crosslinking is able to create a polymer matrix, transforming a viscous solution to a weak gel. This technology prolongs the stability and produces larger, more stable molecules that can mimic the three-dimensional extracellular matrix environment in natural tissues.

Injected soft tissue fillers, containing hyaluronic acid or other substances, induce an inflow of phagocytic neutrophils and mononuclear cells, stimulating macrophages recruiting and fibroblast activation. This reaction occurs because the immune system is unable to enzymatically degrade or phagocytize the injected materials. The inflammatory response to hyaluronic acid fillers, despite the simple composition of these products, is a multifaceted issue. The essential ingredients of these tissue fillers are hyaluronic acid, water, and a cross-linking agent. Each of these elements can influence the induction and development of an inflammatory reaction. Hyaluronic acid itself, despite the fact that it is a natural and common component of the body, may have a proinflammatory effect, in particular, the short chains of this substance. In addition, although unlike other glycosaminoglycans, hyaluronic acid does not bind directly to proteins, so it does not form typical proteoglycans, but it can be an attachment site for other proteoglycans. These ECM-like structures can induce inflammatory responses. High local concentrations of water can also induce an inflammatory process through a local change in osmotic pressure. The last of the three basic components of hyaluronic acid fillers, the cross-linking agent may play a key role in the immunogenic potential of the entire product. The use of polyethylene glycol as a cross-linking agent seems to have a significant immunomodulatory effect, leading to compensating for the proinflammatory effect of the other ingredients of the filler, and even making the entire product exhibit a local anti-inflammatory effect, which is a very desirable effect from the perspective of the safety of filling procedures performed on patients [12].

In the case of hyaluronic acid, cross-linking increases the molecule size, preventing phagocytosis and inducing a protracted cellular response. Granulomatous foreign body reaction (GFBR) after implantation of a biomaterial, or “foreign body” could be considered a normal physiological response from the host to any foreign body. The impossibility to have a normalization of the foreign body response can be related with specific (physicochemical) characteristics of the implanted biomaterial [12].

Due to the increased immune response among patients with autoimmune diseases, including Hashimoto’s disease, such treatments are contraindicated in this cohort of patients [13]. Although hyaluronic acid based hydrogels have been generally considered safe and are well tolerated, recent evidence introduces emerging safety issues related to their immune effects, including delayed hypersensitivity and granulomatous reactions. Degradation of cross-linked hyaluronic acid can reveal traces of cross-linking substances, bacterial proteins, and others. It is known that high-molecular-weight hyaluronic acid exerts primarily an anti-inflammatory effect, while low-molecular-weight hyaluronic acid has a proinflammatory effect and can serve as an endogenous danger signal activating the innate immune system. Theoretically, this independently increases the risk of developing delayed-onset nodules with injection of some family fillers [12].

The very interesting information for all aesthetic medicine practitioners is that too much product, or the wrong choice or type of filler with the wrong properties for a particular area and injection technique, can lead to an immune response. This is an immune response that would not occur with the appropriate use of the product. For example, it is known that nodules of hyaluronic-acid-based filler may appear over time as a result of misuse, incorrect placement of the filler material, displacement of the material, or muscle- or gravity-induced displacement or accumulation and capsular contraction. Choosing the right filler can be crucial; some fillers should not be used in dynamic areas of the face and others have rheological properties that make them unsuitable for superficial administration. This might prolong or even sustain an inflammatory healing response following implantation. The rheology of hyaluronic-acid-based fillers can be modified by a cross-linking agent. When the cross-linking agent is polyethylene glycol (PEG), it guarantees a greater distance between the hyaluronic acid molecules, which induces greater elasticity and other rheological properties of the HA filler cross-linked with PEG [12].

Gradually, exposure or release of these molecules can trigger the immune system. Polyethylene glycol (PEG) as a cross-linking agent for the filler with hyaluronic acid has shown and proven a really high safety profile. Published data suggest that PEG-crosslinked hyaluronic acid hydrogel has excellent chemical and mechanical properties and high biointegration. PEG (PEGDE) used as a crosslinking agent seemed to offer considerable advantages in terms of safety and performance of the hyaluronic-acid-based filer. Both PEG and hyaluronic acid are polymers and their cross-linkage allows the creation of 3D matrices with scaffold structure, constituted by interpenetrated knots and links, thus offering a better integration of the filler into the connective tissue [14]. The technology of hyaluronic acid cross-linking using PEG also guarantees a long duration of the administered hydrogel due to greater resistance to physiological degradation [14]. To date, there have been no reports of granulomas and delayed inflammatory reactions that have been described after the use of PEG cross-linked fillers [15].

Available evidence clearly indicates that it is not possible to predict the effects of glycosaminoglycan-based hydrogels, such as PEG-HA (hyaluronic acid cross linked with polyethylene glycol), on immune cells based solely on their chemical composition, as even slight variations in the chemical structure and component proportions may ultimately have different, and occasionally opposing, effects. Jeong et al. [16] demonstrated that PEGylated hyaluronic acid (PEG-HA) fillers, in vitro, have an high biosafety and [17] reduce immune cell recruitment, the production of reactive oxygen species (ROS), and the expression (mRNA) of proinflammatory cytokines such as tumor necrosis factor (TNF) α and interleukin (IL) 8, both at rest and in stimulation conditions [17]. These findings suggest that PEGylated hyaluronic acid fillers carry a very low risk of immune-mediated adverse effects, particularly granulomatous reaction and associated cellulitic processes, and even induce an anti-inflammatory phenotype in immune cells, which may contribute to the beneficial effects of PEG-HA [15,17].

The aim of this study was to determine the local and systemic effects of Neauvia Stimulate gel in patients suffering from autoimmune thyroid disorders. The Neauvia Stimulate^®^ used in this study is a product that combines pure hyaluronic acid cross-linked with PEG (polyethylene glycol) and microparticles (10–12 µm) of calcium hydroxyapatite in a low concentration (1%). This filler can be considered a “hybrid” filler, completely biocompatible and biodegradable, both with the volumizing effect typical of a cross-linked filler based on hyaluronic acid and with collagenase activity.

## 2. Results and Discussion

### 2.1. Risk Analysis

A total of 15 patients suffering from Hashimoto’s disease at the time of product administration underwent PEGylated HA–Ca (hyaluronic acid cross-linked with polyethylene glycol with a 1% addition of calcium hydroxyapatite molecule size 8–12 microns) filler treatment. A summary of the periprocedural observations is presented in Table 1.

One patient had edema in the first 2 days of follow-up; hyaluronic acid product was palpable in three patients (20%) after 3 days and in one patient after 5 days; none of the patients had tenderness in the application area after 4 days, and none had reddening or the formation of nodules in the treatment area (Table 1). No other events have been recorded during the entire clinical follow-up period (150 days) or reported as of the date of this article.

Anti-TPO and anti-Tg antibodies do not change at 150 days compared to baseline. The mean level of anti-TPO was 257.21 UI/mL before the treatment and 254.14 UI/mL 150 days after the treatment. The mean level of anti-TG was 266.33 UI/mL before the treatment and 267.24 UI/mL 150 days after the treatment (Table 2).

Using the statistical analysis, it was shown that there was no impact of using the tested dermal filler on the levels of anti-TPO and anti-TG antibodies in tested subjects (Table 3).

Correlation for related samples was checked for the null hypothesis “The tested product does not affect level of anti-TPO and anti-TG antibodies”. Results showed positive correlation for this hypothesis (Table 4).

### 2.2. Histopathology Results

**Figure 1:**
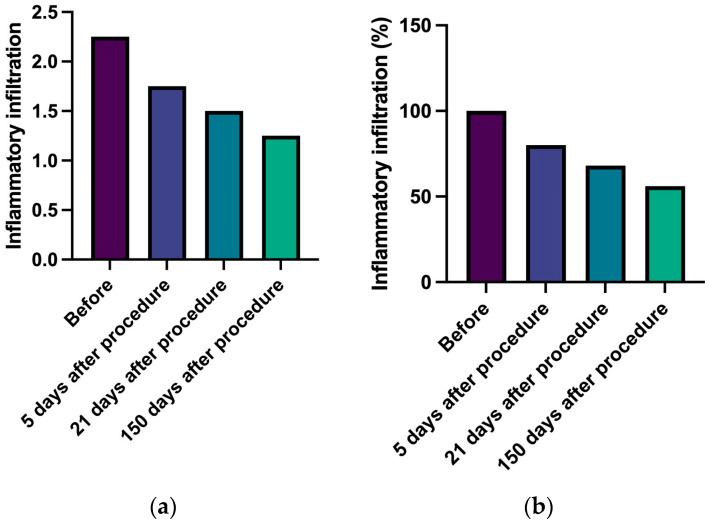
Inflammatory infiltrate in HE staining. (**a**). Inflammatory infiltration change over time—semiquantitive scale (0–4). (**b**). Percentage change in inflammatory infiltration over time.

Local immune infiltrate was studied in five patients at baseline and 5, 21, and 150 days after the injection of PEGylated HA–Ca filler (Figure 1a). As shown in Figure 1b, immune infiltrates progressively decrease from baseline, with a significant variation after 21 days (*p* = 0.00) (Table 5).

**Figure 2:**
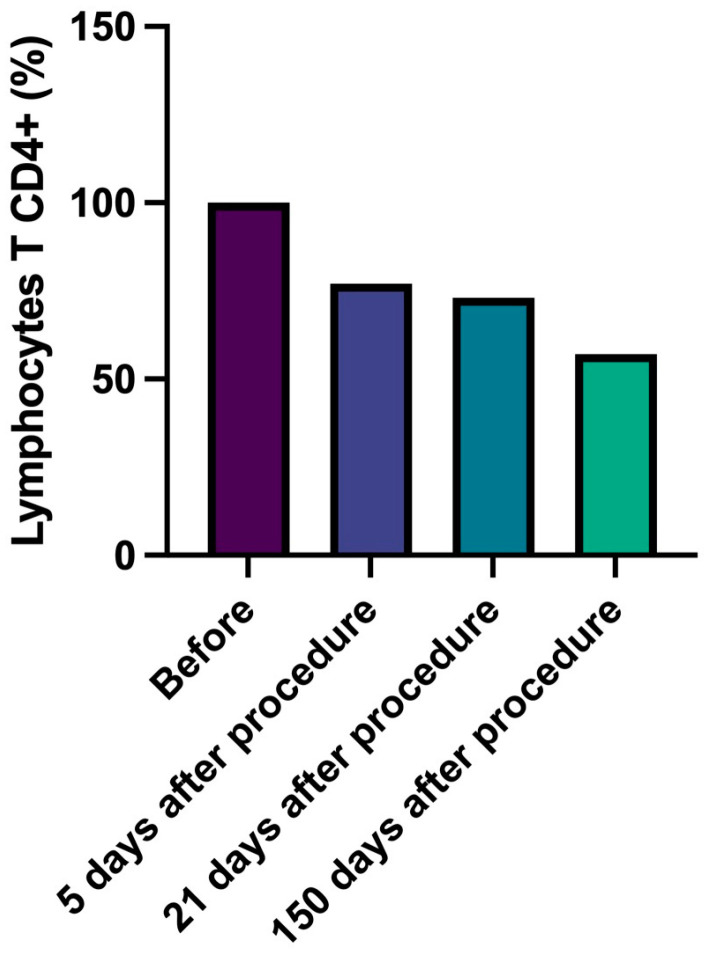
Lymphocytes T CD4 (antigen presenting). Percentage change of number of lymphocytes T CD4+ over time.

**Figure 3:**
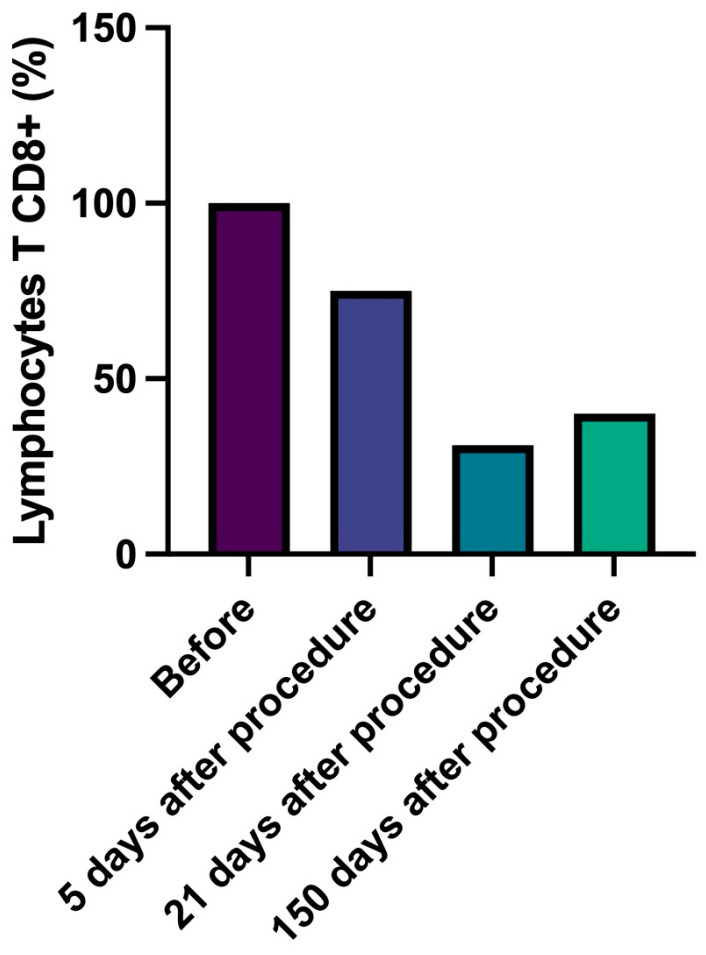
Lymphocytes T CD8 (cytotoxic). Percentage change of number of lymphocytes T CD8+ over time.

**Figure 4:**
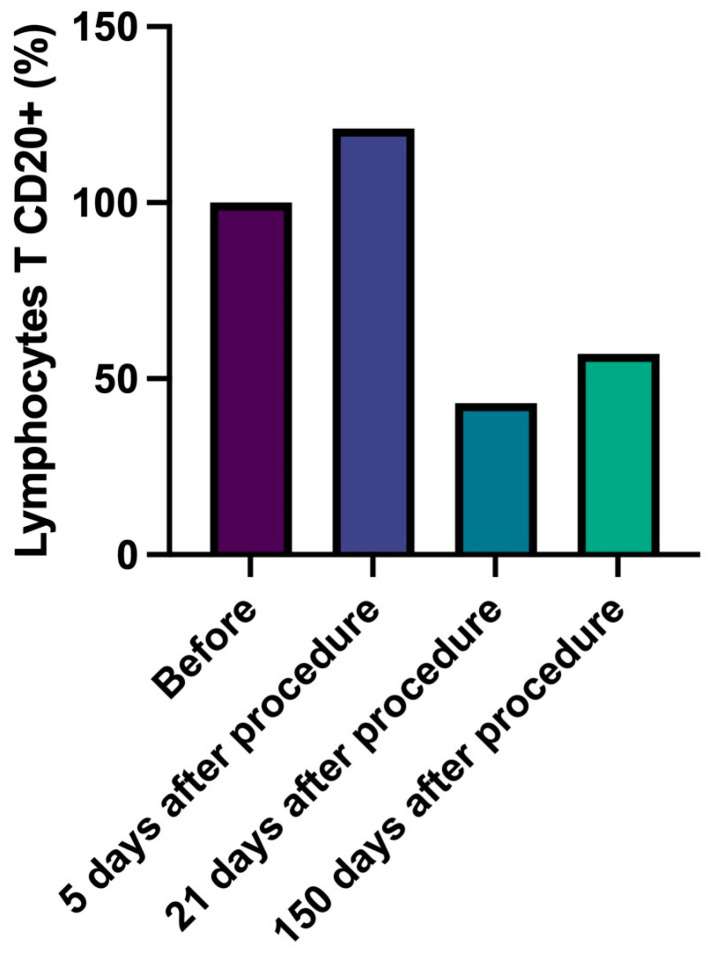
Lymphocytes B CD20. Percentage change of number of lymphocytes B CD20+ over time.

**Figure 5:**
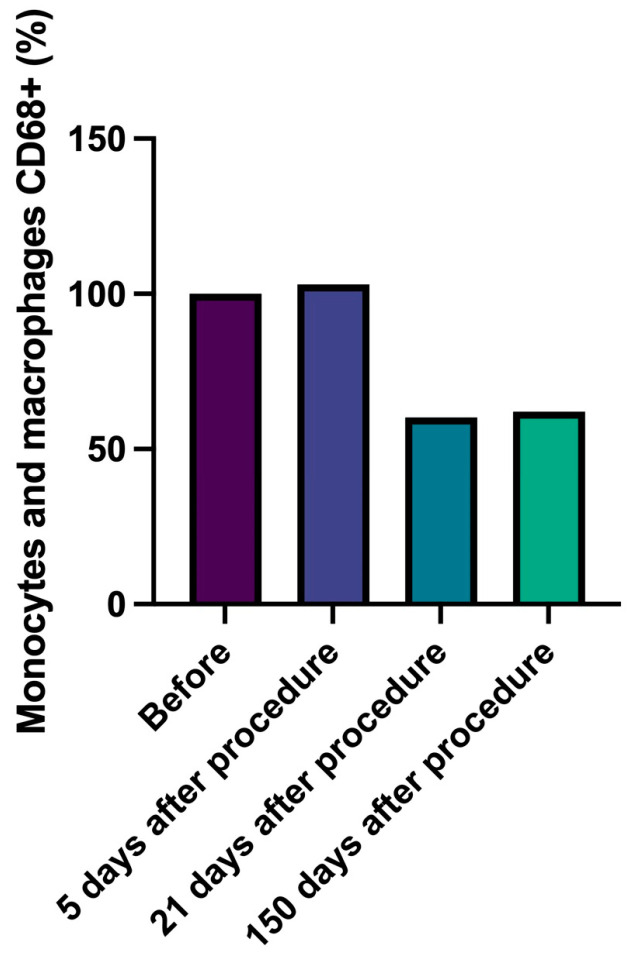
Monocytes and macrophages CD 68. Percentage change of number of monocytes and macrophages CD68+ over time.

In detail, CD4+ T lymphocytes progressively decreased, reaching a number lower than 43% compared to data at 150 days with baseline (Figure 2, Table 6). Similarly, cytotoxic CD8+ T lymphocytes number slightly and progressively decreased during follow-up (delta from baseline to 150 days: −60%) and B lymphocytes (CD20+) number significantly decreased (delta baseline vs. 21 days, −57%, *p* = 0.037) (Figure 3 and Figure 4, Table 7 and Table 8). Innate immune cells CD68+ (macrophages and monocytes) number (Figure 5, Table 9) decreased from baseline at 21 (−39.8%) and 150 days (−38.1%), after an initial slight increase after 5 days.

### 2.3. Discussion

Although hyaluronic-acid-based hydrogels have historically been regarded as safe and well tolerated, recent studies indicate that there may be unexpected safety concerns related to their immunological effects, such as delayed hypersensitivity and granulomatous responses [18,19,20,21,22].

Results concerning the ability of PEGylated hyaluronic acid gel to modulate human immune functions suggest that they carry a very low risk of immune-mediated adverse effects, particularly granulomatous reactions and associated cellulitic processes [15]. In this study, we observed a significant decrease in the overall local inflammatory infiltrate, which reached a frequency significantly lower than before the administration of Neauvia PEGylated fillers. This could be due to the immunomodulatory effect of PEG cross-linking which decreases local inflammation [17]. An increase of cytotoxic T lymphocytes at the site of injection has been described as an undesirable effect of the presence of implants such as hyaluronic-acid-containing fillers, since these lymphocytes mediate the reaction against foreign bodies by MHC class I recognition system [19]. On the contrary, we observed a decrease in T CD8+ lymphocyte number after the procedure. Similarly, an increase of CD68+ cells, which mediate the phagocytosis of foreign bodies, would have been expected. The decrease in the number of antigen-recognizing T lymphocytes (CD4+), cytotoxic T lymphocytes (CD8+), and CD68+ innate immune cells suggests that PEGylated hyaluronic acid gel fillers are not recognized as a foreign body. Cross-linking agents in PEGylated hyaluronic acid gel fillers might have contributed to the high biocompatibility of the incorporated product, which reduces the risk of adverse events related to the immune response, such as the formation of granulomas [15]. An important feature of polyethylene glycol and hyaluronic acid is that they create a matrix with a scaffold structure, a sort of 3D molecular scaffold, that has better integration with the host tissue, gaining a long-lasting effect and better resistance to thermal and mechanical stress. PEGylated hyaluronic acid has a great biointegration and great chemical and mechanical properties compared to other products present on the market cross-linked with other agents instead of polyethylene glycol [14,16,23].

Another arising issue concerned the long-term effects of these fillers, but the biopsy collected eight months after filler injection revealed that the filler was harmoniously integrated into the connective tissues’ collagen fibers, blood and lymphatic vessels, glands, and nerves.

Clinical outcomes of the study, as well as already published data showing no granuloma, foreign body reaction, or other complications, clinically confirm the high safety profile and the high biocompatibility of PEGylated hyaluronic acid fillers with long-lasting results [15].

## 3. Conclusions

The choice of hyaluronic acid fillers cross-linked with polyethylene glycol should be considered justified and safe in the case of patients suffering from Hashimoto’s disease. Reducing the recognition and presentation of the antigen obtained as a result of the immunomodulatory effect of polyethylene glycol has a positive effect on the safety profile both in the context of the use of hyaluronic acid filler itself and the biostimulating effect obtained as a result of the use of calcium hydroxyapatite.

It seems that the limitation of the recognition and presentation of antigens, expressed in a statistically significant decrease in the number of CD4+ and CD8+ T cells in the immediate vicinity of the introduced product, shown in prospective histopathological studies in patients suffering from Hashimoto’s disease, is the basis for the safety of PEGylated hyaluronic acid fillers recorded in long-term retrospective observations also in this group of patients. From the perspective of immunological mechanisms, the subsequent statistically significant reduction in the presence of B lymphocytes as well as monocytes and macrophages is most likely the result of reducing the activity of T lymphocytes.

In this prospective observation of 15 patients, there were no incidents of immune response to the administration of PEGylated hyaluronic acid fillers. In earlier observations from 2014, hyaluronic acid fillers having PEG as cross-linker agent have been introduced in the European market. At that time, several publications were created to evaluate the properties of these fillers. Clinical results support the safety profile of PEG-cross-linked hyaluronic acid, demonstrating no granuloma, foreign body reaction, or other complications over a 3-year period. This is another confirmation of the high safety profile and high biocompatibility, as previously demonstrated in histological and in vitro studies published by Jeong et al. [16].

There are no substantive or registration contraindications to the use of PEGylated hyaluronic acid fillers in patients with Hashimoto’s.

Assessment of the immune effects of hyaluronic-acid-based hydrogel should be, therefore, carefully considered as part of the general safety evaluation of these products. However, sample enlargement, longer follow-up, and the influence of other subject-specific factors should be the subjects of further observations.

## 4. Material and Methods

The study was approved by the local ethic Committee (KB-2/92/2021/ 7 December 2021).

In this prospective study, for safety assessment and to determine the tissue mechanisms accompanying the use of pegylated hyaluronic acid fillers in patients suffering from autoimmune thyroid diseases, a total of 15 Hashimoto’s thyroiditis patients were consecutively recruited (age 26–62 years, female) at Centrum Medyczne dr Kubik entity (a facility of the “K-LAB badania i rozwój” research and development company in Gdynia, Poland) during the period of December 2021–July 2022. Inclusion criteria were age 18–80 years; good general health; diagnosed and treated according to the standards of the Polish Society of Endocrinology; diagnosed active Hashimoto’s disease; consent to participate in the study and acceptance of its course. Exclusion criteria: taking oral retinoids up to 6 months ago; skin and connective tissue diseases (e.g., systemic lupus, collagenopathy, cutaneous porphyria); active or frequently recurring Herpes simplex infection (cold sores); taking medications that may affect the condition of the skin (including tetracycline antibiotics, immunosuppression, i.e., cortisone and its derivatives, anticoagulants, i.e., dipyridamole and coumarin derivatives) for up to 6 months prior; immunodeficiency diseases (including active HIV infection); pregnancy; hypertension; unregulated diabetes; vitiligo or disorders of melanin production, i.e., hypermelanosis; tattoos on treated areas; constant use of anti-inflammatory drugs; previous allergic reactions to local anesthetics and injectable preparations based on hyaluronic acid or calcium hydroxyapatite; tendency to experience scarring.

After signing a written informed consent, all the patients received 2 mL of Neauvia^®^ Stimulate (Matex Lab, Geneve, Switzerland) via the subcutaneous injection technique in the buccal area (both sides of the face) using a blunt 22G cannula. Neauvia Stimulate is a soft tissue filler combining PEG cross-linked hyaluronic acid (26 mg/mL), 1% calcium hydroxyapatite (size 8–12 microns), glycine, and l-proline. The procedures of subcutaneous application of the product were carried out by a team of aesthetic doctors with at least ten years of experience in performing this type of procedure.

All the enrolled patients were evaluated 5, 21, and 150 days after the procedure to assess eventual local adverse reactions. The anti-TPO and anti-TG antibodies levels in peripheral blood on day 0 and day 150 were also tested. Blood samples were tested by an external certified diagnostic laboratory.

In a subgroup of six patients, an additional Neauvia Stimulate subcutaneous procedure in the area behind the ear in the lower retro-ear region (right side, behind the lobus auriculare) was performed using 0.1 mL of the product, a volume which corresponds to the technique used in the area of the cheeks. Then, the complete skin section of this area was taken at baseline and at the 5th, 21st and 150th days after the procedure for histopathological examinations. Skin and subcutaneous tissue samples were collected by a team of surgical and dermatological specialists experienced in collecting material for research related to the pathomorphological assessment of the effectiveness and safety of aesthetic procedures related to the use of various medical devices, including hyaluronic acid fillers. The histopathological examination was performed at the Department of Clinical Pathomorphology, Collegium Medicum in Bydgoszcz, Nicolaus Copernicus University in Toruń, Poland. This assessment was carried out by a team of specialists in the field of clinical pathology experienced in assessing the effect of hyaluronic acid fillers on tissues in in vivo studies. One patient, having a spontaneously evacuating hematoma after the first excision (not related to the given product) in the area where the skin specimen was taken, was excluded due to the potential risk of interference with the histological examination.

Pathomorphological evaluation was performed using hematoxylin–eosin staining and immunohistochemical techniques. Hematoxylin–eosin staining was used to evaluate the overall structure of the tissue by contrasting the cytoplasm and nuclei. Microscopic analysis was performed using the ECLIPSE E400 (NIKON) light microscope equipped with 10× and 20× lenses. The inflammatory infiltrate was assessed on a scale from 0–4 and the frequency of mononuclear cells infiltration (0—not found; 1—up to 25% of the inflammatory infiltrate; 2—between 26–49% of the inflammatory infiltrate; 3—50–75% inflammatory infiltrate; 4—account for 76–100% of the inflammatory infiltrate). An immunohistochemical test was performed to detect CD4+ (CD4+ T lymphocytes), CD8+ (CD8+ T lymphocytes), CD20+ (B lymphocytes) cells, and CD68 + cells (identifying monocytes and macrophages). The test was carried out with the use of mono- or polyclonal rabbit or mouse antibodies (expression of CD4, CD8, CD20, CD68 proteins was assessed as percentage of total mononuclear cells on the scale 0–100%, and CD20 on the scale 0–1).

Statistical analysis was performed using IBM SPSS Statistics software (ver. 28.0.1.0; 2021). The effect of administering the PEGylated HA–Ca hyaluronic acid filler on the level of anti-TPO and anti-TG antibodies, as well as the results of histopathological examinations, were analyzed. For the statistical calculations, the analysis of comparing the means was adopted, and the *t*-test for dependent samples was chosen as the statistical method. For the purposes of correlation tests, the following hypotheses were used:

- Null hypothesis: the tested product does not change the tested parameters;
- Alternative hypothesis: the tested product causes a statistically significant change in the value of the tested parameters.

## Figures and Tables

**Table 1:** Description of local complications and relative frequency.

	Neuvia Stimulate		
	Number of Monitored Patients	Number of Cases	% of Cases
edema up to 2 days	15	1	7%
edema 3–5 days	15	0	0%
edema 6+ days	15	0	0%
palpable product after 3 days	15	3	20%
palpable product after 5 days	15	1	7%
palpable product longer than 10 days	15	0	0%
tenderness in the application area up to 3 days	15	2	13%
tenderness in the application area 4 days and longer	15	0	0%
reddening of the application area up to 3 days	15	0	0%
reddening of the application area 4 days and longer	15	0	0%
the appearance of nodules in the treatment area after 7 days or later	15	0	0%

**Table 2:** Mean levels of anti-TPO and anti-TG antibodies before the treatment and 150 days after the treatment.

Statistics for Dependent Sample					
	Mean	Sample Size N	Standard Deviation	The Standard Error of the Mean	
anti TPO day 0	257.21	15	95.20	25.44	The average level of the variable before the treatment was 257.21, and after 150 days it was 254.14
anti TPO day 150	254.14	15	89.68	23.97	
anti TG day 0	266.33	15	153.84	41.12	The average level of the variable before the treatment was 266.33, and after 150 days it was 267.24
anti TG day 150	267.24	15	148.07	39.57	

**Table 3:** Test of statistical significance of impact of tested product for anti-TPO and anti-TG antibodies.

Test for Dependent Samples									
	Differences in Dependent Samples								
	Mean	Standard Deviation	The Standard Error of the Mean	95% Confidence Interval for the Difference in Means		t	df	Significance *p*	
				Lower Limit	Upper Limit				
anti TPO day 0–anti TPO day 150	3.07	12.80	3.42	−4.32	10.46	0.90	13	0.386	The level of the variable changed on average by 3.07. Conclusions: The analysis showed no effectiveness (*p* > 0.05) of using the preparation on the level of the variable by the subjects after 150 days.
anti TG day 0–anti TG day 150	−0.91	18.78	5.02	−11.75	9.93	−0.18	13	0.859	The level of the variable changed on average by 0.91. Conclusions: The analysis showed no effectiveness (*p* > 0.05) of using the preparation on the level of the variable by the subjects after 150 days.

**Table 4:** *t*-test of correlations results for the hypothesis “The tested product does not affect level of anti-TPO and anti-TG antibodies”.

Correlations for Related Samples				
	Sample Size N	Correlation	Significance *p*	
anti TPO day 0 and anti TPO day 150	14	0.992	0.000	In the *t*-test, the correlation is positive and very strong. The correlation is significant at *p* < 0.05.
anti TG day 0 and anti TG day 150	14	0.993	0.000	In the *t*-test, the correlation is positive and very strong. The correlation is significant at *p* < 0.05.

**Table 5:** Statistical analysis results for inflammatory infiltration.

	Neauvia Stimulate	*p*-Value
Before	100%	
5 days after procedure	80%	0.883
21 days after procedure	68%	0.000
150 days after procedure	56%	0.667

**Table 6:** Statistical analysis results for lymphocytes T CD4+ presence.

	Neauvia Stimulate	*p*-Value
Before	100%	
5 days after procedure	77%	0.694
21 days after procedure	73%	0.038
150 days after procedure	57%	0.951

**Table 7:** Statistical analysis results for lymphocytes T CD8+ presence.

	Neauvia Stimulate	*p*-Value
Before	100%	
5 days after procedure	75%	0.199
21 days after procedure	31%	0.250
150 days after procedure	40%	0.110

**Table 8:** Statistical analysis results for lymphocytes B CD20+ presence.

	Neauvia Stimulate	*p*-Value
Before	100%	
5 days after procedure	121%	0.692
21 days after procedure	43%	0.037
150 days after procedure	57%	0.704

**Table 9:** Statistical analysis results for monocytes and macrophages CD68+ presence.

	Neauvia Stimulate	*p*-Value
Before	100%	
5 days after procedure	103%	0.784
21 days after procedure	60%	0.604
150 days after procedure	62%	0.619

## Data Availability

The data presented in this study are available on request from the corresponding author. The data are not publicly available due to privacy restrictions.

## References

[1] Khunmanee S., Jeong Y. (2017). Crosslinking method of hyaluronic-based hydrogel for biomedical applications. J. Tissue Eng..

[2] Pierre S., Liew S., Bernardin A. (2015). Basics of dermal filler rheology. Dermatol. Surg..

[3] Signorini M., Liew S., Sundaram H., De Boulle K.L., Goodman G.J., Monheit G., Wu Y., Trindade de Almeida A.R., Swift A., Vieria Brz A. (2016). Global Aesthetics Consensus: Avoidance and Management of Complications from Hyaluronic Acid Fillers-Evidence-and Opinion-Based Review and Consensus Recommendations. Plast. Reconstr. Surg..

[4] Bailey S.H., Cohen J.L., Kenkel J.M. (2011). Cosmetic medicine review article: Etiology, prevention, and treatment of dermal filler complications. Aesthetic Surg. J..

[5] Heydenrych I., Kapoor K.M., De Boulle K., Goodman G., Swift A., Kumar N., Rahman E. (2018). A 10-point plan for avoiding hyaluronic acid dermal filler-related complications during facial aesthetic procedures and algorithms for management. Clin. Cosmet. Investig. Dermatol..

[6] Baharvand P., Hormozi M., Aaliehpour A. (2020). Comparison of thyroid disease prevalence in patients with celiac disease and controls. Gastroenterol. Hepatol. Bed Bench..

[7] Wémeau J.-L. (2012). Hashimoto’s thyroiditis (hypertrophic chronić lymphocytic thyroiditis): The centennial of a discovery. Presse Med..

[8] Amino N., Tada H., Hidaka Y., Hashimoto K. (2002). Hashimoto’s Disease and Dr. Hakaru Hashimoto. Endocr. J..

[9] Hiromatsu Y., Satoh H., Amino N. (2013). Hashimoto’s Thyroiditis: History and Future Outlook. Hormones.

[10] Tanda M.L. (2021). Autoimmune thyroid disease. Encyclopedia of Pathology.

[11] Caturegli P., De Remigis A., Rose N.R. (2014). Hashimoto thyroiditis: Clinical and diagnostic criteria. Autoimmun. Rev..

[12] Decates T., Kadouch J., Velthuis P., Rustemeyer T. (2021). Immediate nor Delayed Type Hypersensitivity Plays a Role in Late Inflammatory Reactions After Hyaluronic Acid Filler Injections. Clin. Cosmet. Investig. Dermatol..

[13] Tanda M.L., Piantanida E., Lai A., Lombardi V., Mule D., Liparulo L., Pariani N., Bartalena L. (2009). Thyroid autoimmunity and environment. Horm. Metab. Res..

[14] Zerbinati N., Esposito C., Cipolla G., Calligaro A., Monticelli D., Martina V., Golubovic M., Binic I., Sigova J., Gallo A.L. (2020). Chemical and mechanical characterization of hyaluronic acid hydrogel cross-linked with polyethylen glycol and its use in dermatology. Dermatol. Ther..

[15] Rauso R., Nicoletti G.F., Bove P., Rauso G.M., Fragola R., Lo Giudice G., Zerbinati N. (2021). Clinical Experience with PEGylated Hyaluronic Acid Fillers: A 3-year Retrospective Study. Open Access Maced. J. Med. Sci..

[16] Jeong C.H., Kim D.H., Yune J.H., Kwon H.C., Shin D.M., Sohn H., Lee K.H., Choi B., Kim E.S., Kang J.H. (2021). In vitro toxicity assessment of crosslinking agents used in hyaluronic acid dermal filler. Toxicol In Vitro.

[17] Marino F., Cosentino M., Legnaro M., Luini A., Sigova J., Mocchi R., Lotti T., Zerbinati N. (2020). Immune profile of hyaluronic acid hydrogel polyethylene glycol crosslinked: An in vitro evaluation in human polymorphonuclear leukocytes. Dermatol. Ther..

[18] Owczarzyk-Saczonek A., Zdanowksa N., Wygonowska E., Placek W. (2021). The Immunogenicity of Hyaluronic Acid Fillers and Its Censequences. Clin. Cosmet. Investig. Dermatol..

[19] Bitterman-Deutsch O., Kogan L., Nasser F. (2015). Delayed immune mediatedadverse effects to hyaluronic acid fillers: Report of five cases andreview of the literature. Dermatol. Rep..

[20] Friedman P.M., Mafong E.A., Kauvar A.N., Geronemus R.G. (2002). Safety data of injectable nonanimal stabilized hyaluronic acid gel for soft tissue augmentation. Dermatol. Surg..

[21] Artzi O., Cohen J.L., Dover J.S., Suwanchinda A., Pavicic T., Landau M., Goodman G.J., Ghannam S., Niaimi F.A., van Loghem J.A. (2020). Delayed Inflammatory Reactions to hyaluronic acid fillers: A literature review and proposed treatment algorithm. Clin. Cosmet. Investig. Dermatol..

[22] Artzi O., Loizides C., Verner I., Landau M. (2016). Resistant and recurrent late reaction to hyaluronic acid-based gel. Dermatol. Surg..

[23] Skaalure S.C., Dimson S.O., Pennington A.M., Bryant S.J. (2014). Semi-interpenetrating networks of hyaluronic acid in degradable PEG hydrogels for cartilage tissue engineering. Acta Biomater..

